# Host-functionalization of macrin nanoparticles to enable drug loading and control tumor-associated macrophage phenotype

**DOI:** 10.3389/fimmu.2024.1331480

**Published:** 2024-03-13

**Authors:** Biplab Sarkar, Sean P. Arlauckas, Michael F. Cuccarese, Christopher S. Garris, Ralph Weissleder, Christopher B. Rodell

**Affiliations:** ^1^School of Biomedical Engineering, Science and Health Systems, Drexel University, Philadelphia, PA, United States; ^2^Center for Systems Biology, Massachusetts General Hospital Research Institute, Boston, MA, United States; ^3^Department of Pathology, Harvard Medical School, Boston, MA, United States; ^4^Department of Systems Biology, Harvard Medical School, Boston, MA, United States; ^5^Department of Microbiology and Immunology, Drexel University College of Medicine, Philadelphia, PA, United States

**Keywords:** macrophage, nanoparticle, drug delivery, immunomodulation, cancer immunotherapy

## Abstract

Macrophages are critical regulators of the tumor microenvironment and often present an immuno-suppressive phenotype, supporting tumor growth and immune evasion. Promoting a robust pro-inflammatory macrophage phenotype has emerged as a therapeutic modality that supports tumor clearance, including through synergy with immune checkpoint therapies. Polyglucose nanoparticles (macrins), which possess high macrophage affinity, are useful vehicles for delivering drugs to macrophages, potentially altering their phenotype. Here, we examine the potential of functionalized macrins, synthesized by crosslinking carboxymethyl dextran with *L-*lysine, as effective carriers of immuno-stimulatory drugs to tumor-associated macrophages (TAMs). Azide groups incorporated during particle synthesis provided a handle for click-coupling of propargyl-modified β-cyclodextrin to macrins under mild conditions. Fluorescence-based competitive binding assays revealed the ability of β-cyclodextrin to non-covalently bind to hydrophobic immuno-stimulatory drug candidates (K_eq_ ~ 10^3^ M^-1^), enabling drug loading within nanoparticles. Furthermore, transcriptional profiles of macrophages indicated robust pro-inflammatory reprogramming (elevated *Nos2* and *Il12*; suppressed *Arg1* and *Mrc1* expression levels) for a subset of these immuno-stimulatory agents (UNC2025 and R848). Loading of R848 into the modified macrins improved the drug’s effect on primary murine macrophages by three-fold *in vitro*. Intravital microscopy in IL-12-eYFP reporter mice (24 h post-injection) revealed a two-fold enhancement in mean YFP fluorescence intensity in macrophages targeted with R848-loaded macrins, relative to vehicle controls, validating the desired pro-inflammatory reprogramming of TAMs *in vivo* by cell-targeted drug delivery. Finally, in an intradermal MC38 tumor model, cyclodextrin-modified macrin NPs loaded with immunostimulatory drugs significantly reduced tumor growth. Therefore, efficient and effective repolarization of tumor-associated macrophages to an M1-like phenotype—via drug-loaded macrins—inhibits tumor growth and may be useful as an adjuvant to existing immune checkpoint therapies.

## Introduction

Immune checkpoint therapy has revolutionized the standard of care for treating cancer (1, 2). However, a key limitation of checkpoint-blockade regimens is that only a minority of patients respond to the therapy, as the treatment efficacy is limited by the baseline CD8^+^ T cell clone size (3) and negative feedback from innate immune cells (4). Myeloid cells—in particular, macrophages and neutrophils—in the immuno-suppressive tumor microenvironment support continued growth and immune evasion by tumors (5, 6); furthermore, they have been implicated in the suboptimal response of patients to cancer immunotherapy (7–9). Most tumor-associated macrophages (TAMs) adopt an immuno-suppressive “M2-like” phenotype due to hypoxia, metabolic changes, and epigenetic regulation (10, 11). Fascinatingly, atavistic regression in tumor cells (i.e., onco-fetal reprogramming) (12) and altered levels of extracellular metabolites in the hypoxic microenvironment have been implicated in the upregulation of M2-associated genes in TAMs, such as arginase 1 (*ARG1*) (13), mannose receptor (*MRC1*) (14), and folate receptor beta (*FOLR2*) (15). As these M2-like TAMs enhance tumor cell survival and motility, and because they support tumor growth by stimulating angiogenesis (16), inhibiting their pro-tumor functionality via TAM-targeted therapy is a major clinical need.

Cysteine protease activity is elevated in the highly acidic lysosomes of TAMs (17), negatively affecting their ability to cross-present antigens to CD8+ T cells (18). TAMs express inhibitory immune checkpoints (including PD-L1) and release anti-inflammatory molecules (such as IL-10) that diminish the anti-tumor functionality of cytotoxic CD8^+^ T cells, inhibit CD8^+^ T cell recruitment, and increase the number of T_reg_ cells (11, 19). They can capture anti-PD-1 antibodies from T cell surfaces via F_c_γ receptors, reducing the ability of such antibodies to target the immune checkpoint in T cells (4). Furthermore, TAMs directly contribute to the apoptosis of cytotoxic T cells, attenuating the anti-tumor response of the adaptive immune system. For example, in liver metastases, FasL^+^CD11b^+^F4/80^+^ monocyte-derived macrophages interact with activated antigen-specific Fas^+^CD8^+^ T cells, causing the latter to undergo apoptosis (20). Finally, TAMs form long-lasting antigen-specific synapses with and promote exhaustion in cytotoxic T cells (21, 22). Thus, TAMs inhibit anti-tumor immunity mediated by T cells and reduce the efficacy of lymphocyte-targeted cancer immunotherapy.

Reprogramming TAMs into an M1-like state may be conducive to improving anti-tumor immunity. Moreover, the response of poorly immunogenic tumors to PD-1 blockade is improved by the activation of innate immune cells (23). We and others have previously demonstrated that strategies targeting macrophage polarization are potentially synergistic with immune checkpoint therapy (24, 25). Furthermore, in immunologically “cold” tumors, such as glioblastoma, myeloid cell-targeting nanoparticles delivering immuno-stimulatory molecules reprogram the cells and induce tumor regression (26). Hence, these results are consistent with the finding that alteration of TAM polarization by CSF1R inhibition blocks the progression of glioma (27), tenosynovial giant-cell tumor (28), and lung carcinoma (29). An advantage of nanoparticle-based targeting of macrophages over direct drug-based manipulation is that the former strategy reduces off-target drug effects (24, 30–32).

Nanoparticle composition and surface functionalization affects their ability to target macrophages (33, 34). For example, mannosylated nanoparticles are preferentially internalized by M2-like macrophages, which has been used for therapeutic targeting of MRC1^+^ monocyte-derived macrophages in lung fibrosis (35) and cancer (31). Nanoparticles with similar glucose-based composition (e.g., dextran and cyclodextrin) are likewise preferentially taken up by TAMs, enabling a facile method for imaging (36) and transcriptional reprogramming (24). Specifically, macrins are polyglucose nanoparticles, constructed by crosslinking carboxymethyl dextran with lysine, which have been developed to target macrophages *in vivo* (36–38) and since evaluated in clinical trials as macrophage imaging agents (NCT04843891). However, the capacity of macrins for drug loading and retention is inherently limited, as they are a highly water-swollen polymer networks from which small molecule drugs are rapidly released by diffusion. To overcome this challenge, guest–host chemistry (39–41) provides a facile strategy of sequestering and delivering immuno-stimulatory drugs to the tumor microenvironment via nanoparticles (24, 42). The strategy involves a supramolecular carrier (i.e., host) with a hydrophobic binding pocket, which readily binds to hydrophobic small molecules (i.e., guests) such as drugs (24, 43, 44) or drugs conjugated to appropriate hydrophobic moieties (42, 45).

**Figure 1:**
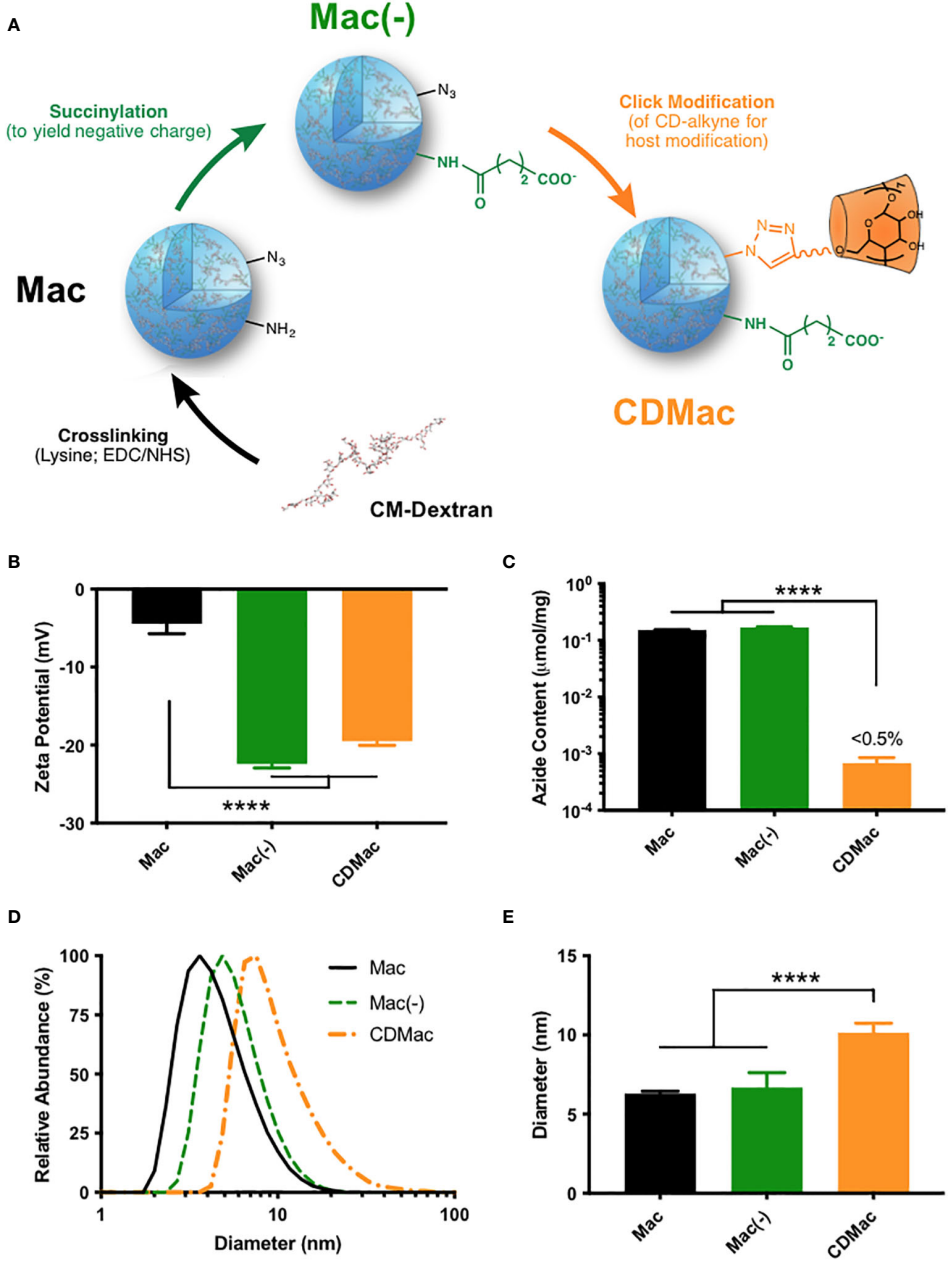
Synthesis of host-modified macrin. **(A)** Lysine crosslinking of carboxymethyl dextran (CM-Dextran) yields macrins (Mac). These nanoparticles were further modified by succinylation **(B)** to yield a negative charge (Mac(-)) and by click-coupling of propargyl-β-cyclodextrin (CDMac, **(C)** consuming available azide groups). **(D, E)** Macrin diameter remained relatively constant during succinylation but significantly increased during host modification. Mean ± s.d., n≥3; ****P<0.0001, ANOVA, Tukey HSD. Schematics in **(A)** have been adapted in part from reference (38). (Keliher et al.; Creative Commons Attribution 4.0 International License).

Here, we introduced β-cyclodextrin (CD) as a host moiety into the dextran network of macrins via click chemistry to construct a macrophage-targeting nanocarrier (referred to as CDMac) (**Figure 1A**). CDMac particles were succinylated to increase their negative charge, aimed at improving *in vivo* residence time. Immuno-stimulatory agents were examined to identify those which could both induce robust macrophage re-education towards an M1-like state and efficiently bind to the nanocarrier through guest–host interactions. If the CDMac nanocarrier itself is preferentially internalized by TAMs, the immuno-stimulatory drugs are likewise delivered via an endosomal route. The goal of this work was to explore host-modified macrins as a tool for therapeutic TAM modulation in anti-cancer therapy. This work has since resulted in vastly improved second-generation nanoparticles with much higher payload capacity, drug synergism, and therapeutic efficacy which are discussed.

## Results

### Design of polyglucose nanoparticles with host modification

Carboxymethyl dextran was crosslinked overnight with lysine using EDC/NHS catalysis to synthesize macrin nanoparticles, according to a previously described method (36–38). The method yielded macrin nanoparticles with a diameter of 6.30 ± 0.25 nm; though, variation in the particle size was found to be readily controllable through synthesis conditions, with a strong dependence on the EDC molar feed ratio, lysine concentration, and polyglucose concentration (**Supplementary Figure S1**). Succinylation of residual amines improved the negative charge of the nanoparticles to –22.4 ± 0.92 mV (**Figure 1B**). To enable host functionalization, β-cyclodextrin was modified with propargyl groups via a 1,6-hexanediamine linker (**Supplementary Figure S2**). Included azide groups in the macrin core structure provided a functional handle for click coupling of propargyl-cyclodextrin to yield cyclodextrin (host)-modified macrins (CDMac). Success of the host conjugation was confirmed by quantification of the free azide content, which was nearly completely eliminated subsequent to the click conjugation (**Figure 1C**). Dynamic light scattering revealed that succinylation did not significantly change the size of macrins; however, host conjugation significantly increased the size of the nanoparticles (10.1 ± 1.04 nm, **Figures 1D, E**) likely attributable to the relatively large quantity and size of the appended groups (>1300 Da).

### *In vitro* activity of immuno-stimulatory drugs and their delivery by host nanoparticles

**Figure 2:**
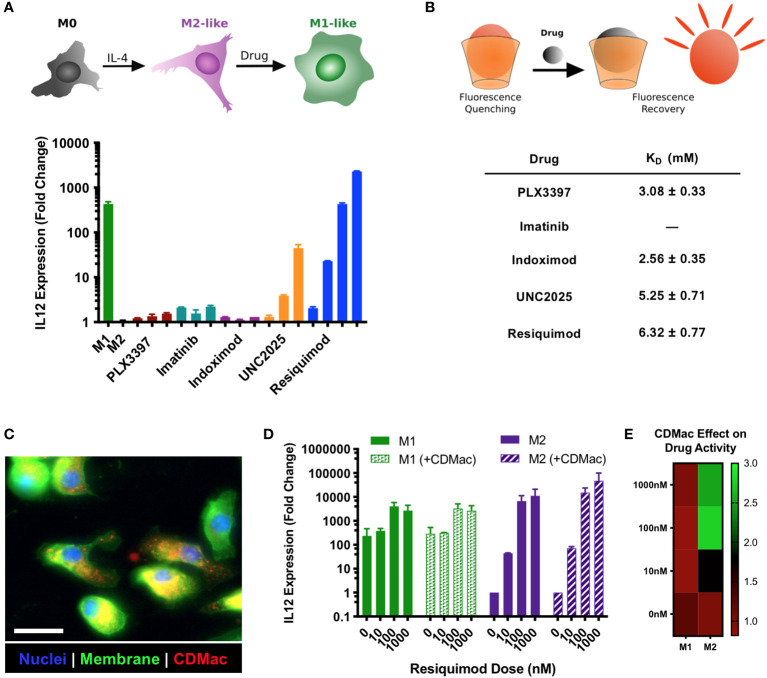
Drug screening and nanoparticle-assisted delivery. **(A)** The capacity for drugs to provoke M2➔M1 re-education was assessed in IL-4 treated BMDMs, using IL-12 expression level as a metric for the M1-like phenotype. Drug concentrations were increased by order of magnitude, with lowest concentrations of 1 µM (indoximod), 100 nM (PLX3397), 10 nM (imatinib, UNC2025), and 1 nM (R848). Mean ± s.d., n=2 per condition. **(B)** The affinity of guest–host complexes was assessed using a colorimetric competitive binding assay to determine the thermodynamic dissociation rate constant, K_D_. Binding for imatinib was not measurable. **(C)** The uptake of CDMac-VT680 was examined by fluorescence microscopy in M2-like (IL-4 treated) BMDMs. Staining: DAPI (nuclei, blue); WGA-AF488 (cell membrane, green); CDMac-VT680 (nanoparticle, red). Scale bar: 25 μm. **(D)** CDMac-assisted delivery of R848 was examined in both M1-like and M2-like BMDMs. Mean ± s.d., n=2 per condition. **(E)** Mean IL-12 expression level with CDMac-assisted delivery, normalized to soluble R848.

For drug selection, we simultaneously evaluated the suitability of potential immuno-stimulatory drugs for M1-like re-education and for guest–host binding with β-cyclodextrin. For the former, murine bone marrow-derived macrophages (BMDMs) were polarized to an M2-like phenotype using IL-4 and subsequently challenged with a set of immuno-stimulatory drugs with the goal of re-educating them to an M1-like phenotype. Owing to literature reports of the drug effects, we examined pexidartinib (i.e., PXL3397; CSF1R inhibitor) (46, 47), imatinib (inhibitor of multiple tyrosine kinases) (48), indoximod (IDO1 inhibitor) (49), UNC2025 (MerTK inhibitor) (50–52), and R848 (i.e., resiquimod; TLR7/8 agonist) (32, 36, 42, 53–57). The expression level of IL-12 was used as a metric for the simple examination of dose-dependent drug effects, as it is a critical mediator of communication between myeloid and lymphoid compartments in the tumor microenvironment and a reliable biomarker of the M1-like state (58). Two drugs showed a high capacity for inducing dose-dependent IL-12 expression: UNC2025 and R848 (**Figure 2A**). Interestingly, the effect of the drugs on the expression levels of M1-associated (*Nos2* and *Il12*) and M2-associated (*Mrc1* and *Arg1*) genes was highly dependent on the presence of IFN-γ (**Supplementary Figure S3**). Notably, only R848 had a high potency and the capacity to elevate IL-12 expression levels even in the absence of IFN-γ, indicating its ability to produce M1-like reprogramming in macrophages. In cross-examining the ability of the drugs to bind to β-cyclodextrin for their delivery, both UNC2025 and R848 had millimolar binding affinity for β-cyclodextrin (**Figure 2B**), indicating their potential for being loaded in the host-modified macrins for cell-targeted delivery. CDMac nanoparticles were rapidly internalized by BMDMs *in vitro* (**Figure 2C**; expanded images available in **Supplementary Figure S4**), which improved the ability of R848 into induce IL-12 expression in M2-like cells by as much as three-fold (**Figures 2D, E**). While these changes in IL-12 expression with and without the CDMac nanocarrier were not statistically significant (two-way ANOVA), *in vitro* studies fail to account for drug pharmacokinetics (e.g., blood half-life, biodistribution) that govern drug efficacy *in vivo*. Our results confirm previous reports that TLR7/8 agonists effectively promote M2-to-M1 re-education (24, 26, 32, 53, 59) and illustrate that nanoparticle-assisted delivery can moderately improve the cellular bioavailability of these drugs even under *in vitro* conditions where cells are continually exposed to extracellular drugs. Furthermore, previous studies have demonstrated that R848 and related derivatives are non-cytotoxic, including towards macrophage cell lines, primary macrophages, and tumor cells (24, 26, 42, 60). Hence, these results indicate the clinical potential of our nano-engineering strategy.

### Nanoparticle biodistribution and effect on TAMs

**Figure 3:**
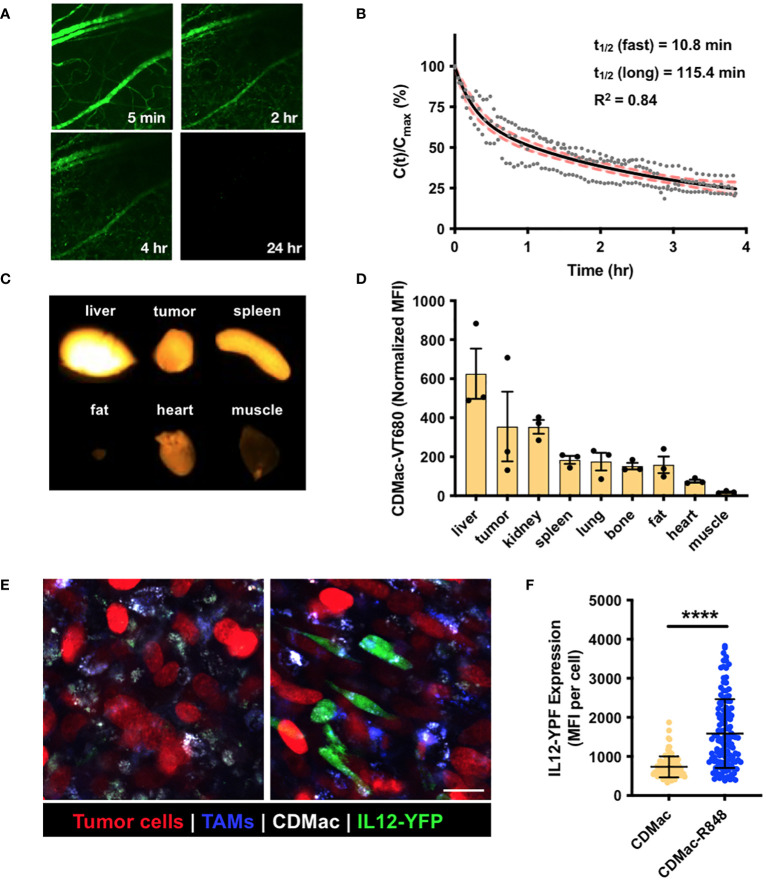
*In vivo* biodistribution and TAM re-polarization. **(A)** Representative fluorescence images of microvasculature in the ear following injection of CDMac-VT680. **(B)** Corresponding quantification of CDMac blood half-life in naïve C57BL/6 mice. Individual data points are presented for n = 3 mice. Data was well fit by a bi-exponential decay, for which the fit curve (black line) and 95% confidence interval (shaded, red) are included. **(C)** Fluorescence reflectance imaging of CDMac-VT680 accumulation in the tumor and organs at 24 h following administration. **(D)** Quantification of the mean fluorescence intensity (MFI), normalized to tissue mass. Mean ± SEM, n=3 mice. **(E)** Representative confocal fluorescence microscopy images of TAMs within tumors 24 h following administration of CDMac (left) or CDMac-R848 (right), each in a single mouse. Scale bar: 20 μm. **(F)** Corresponding quantification of YFP fluorescence. Mean ± s.d., n>100 cells per group across five fields of view per condition; ****P<0.0001, Welch’s *t*-test.

We examined the systemic persistence and biodistribution of CDMac through near-IR fluorescent labeling of the particle (CDMac-VT680). Concentrations in the systemic circulation were assessed by quantification of the fluorescence intensity in microvasculature of the ear in C57BL/6 mice following intravenous injection (**Figure 3A**). Data were best fit to a bi-exponential decay having a long half-life of nearly 2 h (**Figure 3B**), indicative of a prolonged systemic circulation time. We also evaluated the organ-level biodistribution of CDMac in mice bearing a single established MC38 tumor at 24 h after injection, by which time systemic circulation had fully cleared. Apart from the liver, the nanoparticles preferentially accumulated in the tumor tissue (**Figures 3C, D**).

These results are not unexpected, as Kupffer cells and sinusoidal endothelial cells in the liver are known to contribute heavily to nanoparticle clearance from the systemic circulation (61–63), and removal of Kupffer cells is known to improve nanoparticle delivery to tumors (64). Likewise, we determined that CDMac (zeta potential of approximately –20 mV and diameter of ~10 nm) had a long systemic persistence (long half-time of approximately 2 h). Our finding is consistent with the previously established heuristic that relatively large nanoparticles (diameter >5–10 nm) are not readily eliminated by the kidneys (65–67). Indeed, further tuning of biodistribution may be easily accessible, as adjusting catalyst or crosslinker concentrations allowed facile alteration of macrin size and charge (**Figures 1B–E** and **Supplementary Figure S1**), which are known to influence systemic half-life, macrophage uptake, tissue penetration, and ultimate biodistribution (66). However, further exploration of these attributes was not pursued here.

Finally, the cellular biodistribution and effects of the nanotherapeutic on TAMs was directly examined *in vivo* using a dorsal window chamber setup for intravital imaging. We implanted MC38-H2B-mApple tumor cells in IL12-eYFP reporter mice, which were treated with Pacific Blue-dextran as a macrophage-imaging agent to allow for the simultaneous visualization of tumor cells (mApple), TAMs (Pacific Blue) and TAM phenotype (co-expression of IL-12 and eYFP), respectively. At 24 h after treatment by CDMac or CDMac loaded with R848 (CDMac-R848), TAMs readily internalized CDMac, and IL-12-YFP expression level significantly increased after CDMac-R848 administration (**Figures 3E, F**). Interestingly, the variability of IL12-eYFP expression level was high in the CDMac-R848 treatment group (**Figure 3F**). These results were further validated by flow cytometry (**Supplementary Figure S5**). In the tumor microenvironment, TAMs were the predominant immune cell types (nearly 70% of the CD45^+^ population) and the only population of cells observed to uptake CDMac-VT680 to a notable degree. In close agreement with image-based quantification, the mean fluorescence intensity of IL12-eYFP increased by nearly two-fold in TAMs; CD45^+^F4/80^–^CD11c^+^ dendritic cells were also minor contributors to IL-12 production, with a limited number of apparent IL-12^high^ cells.

### Efficacy of drug-loaded nanoparticle treatment *in vivo*

**Figure 4:**
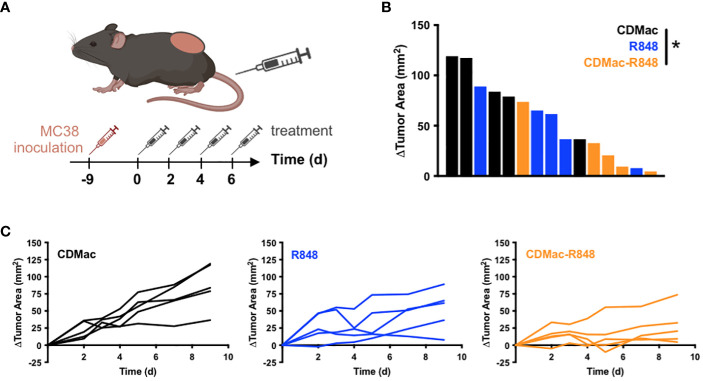
Therapeutic efficacy. **(A)** Schematic overview of treatment regimen. Treatments (CDMac, soluble R848, or CDMac-R848) were administered every other day in mice (n=5 per group) bearing a single established MC38 tumor. **(B)** Waterfall plot of the change in tumor area at day 9, relative to animal baseline. *P<0.05; Kruskal–Wallis, Dunn’s. **(C)** Individual tumor growth curves in response to treatment.

Motivated by observations of successful TAM re-education, therapeutic efficacy of CDMac-R848 was examined in C57BL/6 mice bearing a single established MC38 tumor. Owing to the unknown potential for re-educated M1-like TAMs to revert to a M2-lke phenotype, a repeated administration regimen was used (**Figure 4A**). While individual tumor response to free drug treatment was highly variable (**Figures 4B, C**), CDMac-R848 treatment homogenized treatment response and led to a qualitatively more consistent reduction in tumor volumes over time. Relative to vehicle controls, mean tumor growth was significantly reduced by CDMac-R848 but not by the free drug, including at the study endpoint on day nine post-treatment (**Figure 4B**, **Supplementary Figure S6**).

## Discussion

TAMs are highly abundant in many solid tumors (68) and constituted 70% of the total tumor immune cell population in our study. Macrophage polarity in the tumor microenvironment spans a wide gamut, contributing to a high level of transcriptomic diversity (9). Matrix stiffening (69), efferocytosis of apoptotic cells (70, 71), and altered levels of metabolites (10) during tumor progression shift the phenotypic balance of TAMs toward a pro-tumor M2-like phenotype. Myeloid cells, particularly macrophages, orchestrate innate immune pathways, are prone to nanoparticle uptake, and possess phenotypic plasticity, making them an attractive target for nanoparticle-mediated drug delivery (24, 30–32). Immuno-stimulation of TAMs via pH-gated nanoparticles (17), drug-carrying nanoparticles (26), or hydrogels (72, 73) promotes phagocytosis of tumor cells by macrophages and supports an anti-tumor T cell response (74). Our results suggest that cyclodextrin-modified macrins enabled targeted drug delivery to TAMs, reprogramming them into a M1-like phenotype. Macrins are known to preferentially target macrophages (36), and TAMs were the most abundant immune cells in the tumor (according to our flow cytometry results). In combination, these two aspects explain the propensity of CDMac to preferentially accumulate in the tumor.

Interestingly, targeting cell-surface receptors on macrophages provides an avenue to reprogram macrophage phenotype. For example, a monoclonal antibody targeted to the scavenger receptor MARCO was shown to reprogram TAMs into M1-like macrophages, inducing anti-tumor activity (in models of breast cancer, colon carcinoma, and melanoma) and enhancing the efficacy of immune checkpoint therapy (75). Furthermore, engaging MRC1 via synthetic mimics of amphipathic host-defense peptides induces endocytosis, phagosome/lysosome formation, and autophagy, reprogramming M2-like TAMs to anti-tumor M1-like state (76). An alternative strategy is enabled by the propensity of macrophages to internalize nanoparticles via endosomes, which contain toll-like receptors (TLRs) such as TLR7/8 (77). Activation of such intracellular TLRs can polarize TAMs into an M1-like state and lead to the production of pro-inflammatory cytokines such as IL-12, via NF-κB signaling (24, 53, 54, 56, 78). In our study, the toll-like receptor agonist R848 demonstrated a potent ability to induce M1-like reprogramming in M2-like macrophages. Enhancement of this effect by nanoparticle-assisted delivery may be aided by the fact that R848 is an TLR7/8 agonist, effectively targeted to these receptors in the endosome upon internalization.

Upregulating NF-κB signaling promotes M1-like polarization in TAMs in ovarian cancer (79) and glioblastoma (74), thwarting tumor progression. Upregulation of NF-κB signaling is critical for myeloid expression of IL-12 (80), a key link between the innate immune activation and adaptive immune response (81). IL-12 is also known to potently induce anti-tumor immunity (58, 82). Specifically, IL-12 produced by M1-like macrophages and dendritic cells (DCs) contribute to natural killer (NK) cell activation (early stage) and conversion of naïve CD4^+^ T cells into T_H_1 cells (late stage) (81). Interestingly, M2-like TAMs indirectly diminish the cytotoxic function of CD8^+^ T cells, in part, by releasing IL-10 that suppresses IL-12 expression in intra-tumoral dendritic cells (19). Hence, increasing IL-12 production by M1-repolarized TAMs may partly restore the anti-tumor activity of CD8^+^ T cells. Our results revealed that the macrophage-directed delivery of drugs, such as R848, via CDMac nanoparticles significantly increased IL-12 expression level in TAMs, potentially explaining the *in vivo* anti-tumor functionality of drug-loaded nanoparticles. Interestingly, the role of IL-12 somewhat mirrors the activity of IFN-γ in the crosstalk between myeloid cells and lymphocytes. IFN-γ produced by activated NK cells, T_H_1 cells, and CD8^+^ T cells is sensed by monocytes and dendritic cells (DCs), adding to the population of M1-like macrophages and activated DCs that produce IL-12 (58). In our study, exogenous IFN-γ improved the M1-reprogramming capability of drugs such as R848 and UNC2025.

We would like to note two cautionary points. First, the tumor microenvironment varies in different organs, and TAMs may not always neatly conform to an M2-like state (9, 83–86). For example, FOLR2^+^ macrophages in human breast tumors interact with CD8^+^ T cells and promote anti-tumor immunity (86); in contrast, in hepatocellular carcinoma, FOLR2^+^ macrophages participate in immunosuppressive interactions with T_reg_ cells (15). Second, upregulating M1-associated signature does necessarily improve the efficacy of immune checkpoint therapies. For example, pharmacological inhibition of ARG1 in TAMs does not synergize with anti-PD-1 therapy, as anti-PD-1 therapy itself decreases the abundance of ARG1^+^ TAMs while increasing that of ARG1^–^ TAMs (13). Thus, the mechanistic basis of macrophage targeting needs to be clear and context-specific.

Our study, which was conducted in 2017, was not without limitations but ultimately led to drastic improvements in the overall approach and material design. First, the macrin nanoparticles had a limited number of available host moieties, as most of the nanoparticle was comprised of linear dextran chains with no hosting capability. Second, although we demonstrated that drug-loaded macrins induce M1-like programming in macrophages, we did not directly evaluate tumor cell killing by macrophages or by lymphocytes activated by IL-12. Third, a comprehensive analysis of myeloid cell targeting in the tumor microenvironment was not conducted here and may be a useful topic of further research. There remains room for further understanding the mechanistic basis of macrophage-activating therapeutics, including in the context of mono- or adjuvant therapies. Particularly, antigen-specific anti-tumor immune response potentially triggered by re-polarized TAMs was not directly studied in this work and has recently emerged as an interesting therapeutic modality (17, 32).

In work that arose as a direct result of this study, we improved the hosting capacity of the platform by designing cyclodextrin nanoparticles (CDNPs) which do not contain any dextran molecules; this adaptation was allowed by the direct crosslinking of succinylated β-cyclodextrin by lysine. Furthermore, we did demonstrate that M1-like reprogramming of TAMs is a viable strategy for cancer immunotherapy, including by synergy with anti-PD1 therapy and with dependence on involvement of the adaptive immune compartment (24) or independent of T cells in the context of glioma (26). Hence, the lessons we learned from the current study directly resulted in several impactful published articles involving the CDNP platform, which have included the delivery of alternative therapeutics for cancer and other diseases (43, 44, 87), the direct modification of drugs by host groups to enable improved drug binding (42), and multi-drug delivery for the formation of even more highly activated myeloid cells (74, 88).

Therefore, macrophage engineering using drug-loaded nanoparticles has an underexplored potential to reprogram the immuno-suppressive tumor microenvironment and potentiate favorable response toward immune checkpoint therapy, removing a critical roadblock for clinical translation. Emerging avenues—such as the introduction of phagocytosis-triggering CARs (89–91), metabolic reprogramming (92), targeting lysosomal function (17, 18), induction of trained immunity (93), and synergistic targeting of complementary immuno-stimulatory pathways in TAMs (74, 88)—are worth exploring to realize the potential of *in vivo* macrophage engineering to treat solid tumors and metastases.

## Conclusions

Here, we developed a nanotherapeutic platform that is suitable for the delivery of a variety of small molecule immuno-stimulatory drugs into TAMs, taking advantage of the exceptional macrophage avidity of macrins and extending their utility through the addition of β-cyclodextrin moieties to enable the guest–host inclusion of hydrophobic drugs. The resulting macrophage-targeted drug vehicle (CDMac) non-covalently sequestered a potent immuno-stimulatory drug candidate (R848) that demonstrated a pronounced ability to induce M1-like programming. Intravital microscopy in IL12-eYFP reporter mice revealed a two-fold enhancement in mean YFP fluorescence intensity in macrophages targeted with R848-loaded CDMac, relative to vehicle controls alone, indicating M1-like reprogramming of TAMs *in vivo*. While free drug controls were not included in these imaging studies, nanoparticle-assisted delivery significantly enhanced drug effects relative to the free drug *in vitro* and better controlled tumor growth in mice. Our study indicates that *in vivo* macrophage re-polarization via drug-loaded nanoparticles is a viable strategy to target the immuno-suppressive tumor microenvironment, potentially improving the efficacy of co-administered lymphocyte-targeted immunotherapeutics.

## Materials and methods

### Materials

Unless otherwise indicated, solvents and general reagents were procured from Sigma-Aldrich and used without additional purification. Pharmacological drugs were purchased from Selleckchem (imatinib mesylate, PLX3397, indoximod, and R848) and MedchemExpress (UNC2025). Carboxymethyl dextran (10 kDa, 5% carboxylated) was purchased from TdB. Amino-dextran (500kDa, Thermo Fisher) was fluorescently labeled by Pacific Blue (label concentration: 40.1 ± 2.6 nM mg^-1^) as previously described for intravital imaging (94). For all experiments, water was purified using a MilliQ filtration system (Waters).

### Nanoparticle synthesis

Macrins (Mac) were prepared as previously described (38). Nanoparticles were formed by the simultaneous reaction of carboxymethyl dextran with azido-acetic N-hydroxysuccinimidyl ester (to introduce azide groups for later click-coupling) and *L-*lysine (as a crosslinker), catalyzed by N-(3-dimethylaminopropyl)-N’-ethlycarbodiimide hydrochloride (EDC; Thermo Fisher, 22980) and N-hydroxysuccinimide (NHS). Resulting macrins were recovered by precipitation from ice cold ethanol and further purified by size-exclusion chromatography. Macrins were further modified to produce a negative charge through succinylation (Mac (-)). Macrin stocks (25 mg/mL) were prepared in MES buffer (50 mM, pH 6.0), to which triethyl amine (1 µL per mg macrin) and excess succinic anhydride (100 mg per 1 mg macrin, dissolved in DMSO at 0.5 g/mL) were added. The reaction was vigorously mixed overnight prior to purification by elution through a PD-10 column (GE Healthcare, 17-0851-01) and subsequent concentration by centrifugal filtration (10 kDa MWCO, Sigma, UFC501096).

To allow for click-coupling of the host group, CD was modified by alkynes through a 1,6-diaminohexane linker (**Supplementary Figure S1**). Briefly, aminated CD (6-(6-aminohexyl)amino-6-deoxy-β-cyclodextrin) was prepared via the tosylate intermediate (6-*o*-monotosyl-6-deoxy-β-cyclodextrin), according to published protocols (95). Subsequently, aminated CD (500 mg, 1.0 eq.) was reacted with propargyl-N-hydroxysuccinimidyl ester (114 mg, 1.25 eq.) by dissolution in anhydrous DMF (4 mL) followed by the dropwise addition of triethylamine (0.21 mL, 2.5 eq, diluted in 4 mL DMF) under dry argon. After overnight reaction at 30°C, a second bolus of propargyl-N-hydroxysuccinimidyl ester (50 mg, dissolved in 1 mL DMF) was added with subsequent reaction for 24 h to ensure reaction completion. The reaction solution was concentrated to 2 mL under reduced pressure and precipitated twice from a ten-fold excess of ice-cold acetone to yield a white powder that was washed twice each by ice cold acetone and diethyl ether. The product was dried under vacuum overnight prior to ^1^H-NMR in DMSO-d_6_. For click-coupling of macrins with the propargyl-modified CD, succinylated macrins (2 mL, 10 mg/mL) were combined with cupric sulfate (1 mL, 100 mM in water), sodium ascorbate (1 mL, 100 mM in water), and propargyl-CD (1.2 mol excess, relative to azide). After vigorously stirring overnight at 40°C, the modified macrins (CDMac) were recovered by elution through a PD-10 column and subsequent concentration by centrifugal filtration.

For imaging studies, nanoparticles were fluorescently labeled by dissolution at 20 mg/mL in carbonate buffer (0.1 M, pH 8.5) prior to addition of VivoTag 680 XL (PerkinElmer, 1.0 mg/mL in anhydrous DMSO) at a final concentration of 50 μM. The reaction was allowed to proceed for 3 h at room temperature to yield CDMac-VT680. For final purification, all crude nanoparticle preparations were re-dissolved in water, concentrated by centrifugal filtration, washed repeatedly by water, and lyophilized. The final products (Mac, Mac (-), CDMac, and CDMac-VT680) were re-dissolved at a concentration of 10 mg/mL and stored at –20°C until further use.

### Nanoparticle characterization

Particle size was determined using dynamic light scattering (DLS; Malvern, Zetasizer APS; n=3 per group) at a typical concentration of 4.0 mg/mL in 100 mM PBS. Zeta potential was determined at 10 μg/mL in 10 mM PBS (Malvern, Zetasizer ZS; n=4 per group). For quantification of azide content, nanoparticle samples (20 µL, 10 mg/mL, n=4 per group) were combined with cupric sulfate (10 µL, 100 mM in water), sodium ascorbate (10 µL, 100 mM in water), and 5-propargyl-fluorescein (10 µL, 10 mM in DMSO). After shaking at 40°C for 2 h, nanoparticles were recovered by elution through a PD-10 column and subsequent concentration by centrifugal filtration. Absorption at 490 nm (Nanodrop) was used to determine the fluorophore concentration conjugated on the nanoparticle, based on the Beer–Lambert equation (*A* = *γbc*; where *A* is the absorbance, *γ* is the molar absorptivity [80,000 M^−1^ cm^−1^ for 5-propargyl-fluorescein], *b* is the path length, and *c* is the concentration). For CDMac-VT680, absorption at 668 nm (Nanodrop) was used to determine the label concentration (18.9 ± 0.36 nol/mg) using the Beer–Lambert equation, (A = *ε*bc, where A is the absorbance, *ε* is the molar absorptivity 210,000 M^-1^cm^-1^, and c is the concentration).

### Guest–host affinity measurement

Analysis of drug affinity for CD was performed by a standard colorimetric competitive binding assay (96), which was internally optimized to improve assay sensitivity by using a 200 µM concentration of phenolphthalein and a 5 mM concentration of CD. For all experiments, stocks of phenolphthalein were freshly prepared in carbonate buffer (125 mM, pH 10.5). The decrease in absorbance at 550 nm due to guest–host complexation of phenolphthalein with CD and absorbance recovery due to competitive drug binding were measured (Tecan, Spark), accounting for sample dilution by drug addition. The thermodynamic dissociation rate constant, K_D_, was determined by the addition of increasing concentrations of drugs and fit to a one-site competitive inhibition model.

### Cell culture

The MC38 mouse colon adenocarcinoma cell line was provided by M. Smyth (QIMR Berghofer Medical Research Institute) and subsequently modified by stable transfection of the H2B-Apple reporter to yield an MC38-H2B-mApple cell line employed in intravital microscopy studies, as previously described (4). MC38 and MC38-H2B-Apple reporter cells were cultured in Iscove’s DMEM supplemented with 10% fetal bovine serum, 1% penicillin/streptomycin, and 1% L-glutamine. Bone marrow cells were isolated from the surgically resected femur and tibia of naive C57BL/6 mice as previously described (97). Once plated at a density of 2.5 × 10^6^ cells/mL in 48-well (Corning, 3527, for PCR analysis) or 1 × 10^6^ cells/mL in optical-bottom 384-well plates (Thermo Fisher, 142761, for image analysis), bone marrow-derived macrophages (BMDMs) were derived in Iscove’s Modified Dulbecco’s Medium supplemented with 10% heat-inactivated fetal calf serum, 100 IU penicillin, 100 μg/mL streptomycin and 10 ng/mL M-CSF (PeproTech, 315-02) with media replenished every two days. All cells were maintained in the indicated medium at 37°C and 5% CO_2_ with regular mycoplasma screening for cell lines.

### *In vitro* phenotyping

For transcriptional analysis of BMDMs, media was replenished with M-CSF-free media on day 7 including supplementation by pharmacologic drugs at the prescribed concentrations. Standard reference phenotypes were included as controls: M2-like (10 ng/mL IL-4) and M1-like (100 ng/mL LPS, 50 ng/mL IFN-*γ*) phenotypes. At 24 h post-treatment, RNA was isolated (QIAGEN, 74106), reverse transcribed (Thermo Fisher, 4368814), and subject to qPCR using Taqman Fast Advanced Master Mix and probes for *Hprt* (Mm01545399_m1), *Il12b* (Mm01288989_m1), *Nos2* (Mm00440502_m1), *Cd80* (Mm00711660_m1), *Arg1* (Mm00475988_m1), and *Mrc1* (Mm01329362_m1). Data is expressed as a fold change in gene expression level using the ΔΔCt method (98), relative to the *Hprt* and M2-like controls.

### Pharmacokinetic and biodistribution analysis

For imaging of nanoparticle uptake *in vitro*, M2-like BMDMs were treated with CDMac-VT680 (100 μg/mL) for 4 h. Cells were subsequently washed by PBS, fixed with paraformaldehyde (4%, 30 min, 37°C), and stained (cell membrane: 5.0 μg/mL Alexa Fluor 488 wheat germ agglutinin, Thermo Fisher; nuclei: DAPI, Invitrogen) for 15 min at room temperature. Plates were washed and subsequently imaged on a custom high-content screening microscope (Olympus).

The blood half-life of CDMac-VT680 (1 mg, 100 μL saline, administered by tail vein injection) was determined by confocal fluorescence microscopy of vessels in the ear of C57BL/6 mice (n=3). Time-lapse images were acquired continually during injection and over the first 4 h, with follow-up image acquisition at 24 h. Per mouse, multiple regions of interest were identified within the labeled vasculature and the mean fluorescence intensity determined as a function of time. Images were uniformly background subtracted prior to quantification, and data were normalized to their peak intensity and fit to a bi-exponential decay. At 24 h following injection, CDMac-VT680 biodistribution was examined in the C57BL/6 mice bearing established MC38 tumors. Surgically resected tissues were washed in PBS, massed, and imaging performed using a small animal imaging system (OV110, Olympus). Acquisition included brightfield imaging to identify regions of interest and fluorescence reflectance imaging (*λ*_ex/em_ = 620–650/680–710 nm). The integrated fluorescence density was determined for each tissue sample, normalized to tissue mass, and background of tissues from a vehicle-treated control mouse subtracted to account for tissue autofluorescence.

### Intravital microscopy

To examine the cellular biodistribution and induction of IL-12 *in vivo*, intravital imaging was performed using dorsal skinfold window chambers installed on p40-IRES-eYFP IL-12 reporter mice inoculated with MC38–H2B-mApple tumors using methods similar to those previously described (4, 99). Mice received CDMac-VT680 (1 mg, 100 μL saline) or CDMac-VT680 + R848 (1 mg CDMac-VT680 + 2.0 mg/kg R848, 100 μL saline) each prepared in 50 μL sterile saline by tail vein injection 24 h prior to imaging. Macrophages were likewise labeled by Pacific Blue–dextran. Images were pseudo-colored and processed in FIJI (100) by adjusting brightness/contrast, creating z-projections of image stacks, and performing a rolling ball background subtraction. For quantification of IL-12 expression level, the sum of YFP, Pacific Blue, and VT680 channels were segmented by automated thresholding using the Rényi Entropy method to generate a mask and corresponding ROIs for individual macrophages. The mean fluorescence intensity was determined for YFP within each ROI. For all confocal imaging, acquisition was performed using a FV1000MPE (Olympus). Pacific Blue, GFP/YFP, mApple, and VT680 were sequentially excited using 405-, 473-, 559-, and 635-nm diode lasers and BA430-455, BA490-540, BA575-620, and BA655-755 emission filters with SDM473, SDM560, and SDM640 beam splitters.

### Flow cytometry

To further examine the cellular biodistribution and induction of IL-12 in the tumor microenvironment, CDMac-VT680 (1 mg, 100 μL saline) was administered by tail vein injection in IL-12-eYFP reporter mice bearing established MC38 tumors 48 h prior to examination. Tissues were minced, incubated in RPMI containing 0.2 mg/mL collagenase I (Worthington Biochemical) for 30 min at 37°C and then passed through a 40 μm filter. Red blood cells were lysed using ACK lysis buffer (Thermo Fisher), Fc receptors blocked by anti-CD16/32 (BioLegend, clone 93), and cells stained in phosphate buffered saline containing 0.5% BSA and 2 mM EDTA with fluorochrome labeled antibodies against CD45 (eBioscience, 30-F11), CD11c (BioLegend, N418), Ly6G (BioLegend, 1A8), F4/80 (BioLegend, BM8), and 7-AAD. Samples were run on a LSR II flow cytometer (BD) and analyzed in FlowJo v.8.8.7 (Tree Star, Inc.) to identify macrophages (CD45^+^Ly6G^–^F4/80^+^), dendritic cells (CD45^+^F4/80^–^CD11c^+^), and other immune cells (CD45^+^F4/80^–^CD11c^–^).

### Animal models

Animal research was conducted in compliance with the Institutional Animal Care and Use Committees at Massachusetts General Hospital (MGH). Unless otherwise stated, experiments were performed using C57BL/6 mice (female, 6–8 weeks of age at experiment initiation; Jackson, 000664). Intravital examination of CDMac-VT680 and IL-12 expression was concurrently performed using p40-IRES-eYFP-IL-12 reporter mice (Jackson, 015864). Tumor growth studies were initiated by intradermal injection (2 × 10^6^ MC38 cells, 50 μL PBS). Treatment groups were assigned such that body weight and tumor size were normalized across groups at baseline once tumors reached an established size of 25 mm^2^ (100 mm^3^). Mice were treated every other day tail vein injection of R848 (2.0 mg/kg), CDMac (1 mg), or equivalent dosing of CDMac-R848, each in 100 μL saline. Tumor growth was monitored by caliper measurement (A = length × width) and values are reported following normalization to baseline.

### Data analysis

Image analysis was performed in FIJI (100). Statistical analyses were performed using GraphPad Prism 9.5.0. Unless indicated, data are presented as mean ± standard deviation (SD). Statistical significance was determined by ANOVA with *post hoc* Tukey’s honestly significant difference (HSD) test. For tumor growth, temporal analysis was made by Friedman’s test and comparison at set time points was performed by Kruskal–Wallis test with *post hoc* Dunn’s test. Significance cut-off was set at P < 0.05.

## Data availability statement

The raw data supporting the conclusions of this article will be made available by the authors, without undue reservation.

## Ethics statement

The animal study was approved by Institutional Animal Care and Use Committees at Massachusetts General Hospital. The study was conducted in accordance with the local legislation and institutional requirements.

## Author contributions

BS: Visualization, Writing – original draft, Writing – review & editing. SA: Data curation, Formal analysis, Investigation, Methodology, Visualization, Writing – review & editing. MC: Data curation, Formal analysis, Investigation, Methodology, Writing – review & editing, Visualization. CG: Data curation, Formal analysis, Investigation, Methodology, Visualization, Writing – review & editing. RW: Conceptualization, Funding acquisition, Project administration, Resources, Supervision, Writing – original draft, Writing – review & editing. CR: Conceptualization, Data curation, Funding acquisition, Investigation, Methodology, Supervision, Writing – original draft, Writing – review & editing.
