# Supplementary material for: Host-functionalization of macrin nanoparticles to enable drug loading and control tumor-associated macrophage phenotype
Source: Front Immunol. 2024 Mar 13;15:1331480. doi: 10.3389/fimmu.2024.1331480 (PMC10965546; doi:10.3389/fimmu.2024.1331480)
Supplement: Supplementary file 1 [file DataSheet_1.pdf]

Supplementary Material

Host-Functionalization of Macrin Nanoparticles to Enable Drug Loading and Control Tumor-Associated Macrophage Phenotype

Biplab Sarkar[1], Sean P. Arlauckas[2], Michael F. Cuccarese[2], Christopher S. Garris[2,3], Ralph Weissleder[2,4], Christopher B. Rodell[1,5]*

[1]School of Biomedical Engineering, Science and Health Systems, Drexel University, Philadelphia, PA, USA

[2]Center for Systems Biology, Massachusetts General Hospital Research Institute, Boston, MA, USA

[3]Department of Pathology, Harvard Medical School, Boston, MA, USA

[4]Department of Systems Biology, Harvard Medical School, Boston, MA, USA

[5]Department of Microbiology and Immunology, Drexel University College of Medicine, Philadelphia, PA, USA

*** Correspondence:**
Christopher B. Rodell, PhD
christopher.b.rodell@drexel.edu

Supplementary Methods:

To examine the capacity to modify macrin size, such as to prevent renal clearance and prolong blood half-life, alternative synthesis conditions were explored. Macrins were prepared by the reaction of carboxymethyl dextran (100 mg, 1.0 eq. carboxylate, 1.25 to 20.0 $\%_{w/v}$), N-(3-dimethylaminopropyl)-N′-ethlycarbodiimide hydrochloride (EDC; Thermo Fisher, 22980; 5 to 20.0 eq. to carboxylate), and N-hydroxysuccinimide (Sigma, 0.5 eq. to EDC), which were combined in a scintillation vial and dissolved in MES buffer (50 mM, pH 6.0). After stirring for 30 min at room temperature, *L*-lysine (0.25 to 8 eq. to carboxylate) was added by dropwise addition prior to overnight reaction. Unless otherwise specified, synthesis parameters were fixed at 5%w/v dextran, 10 eq. EDC, and 1 eq. lysine. Resulting macrins were recovered by the addition of brine (0.05 volumetric equivalents) and precipitation from a tenfold excess of ice-cold ethanol prior to purification by PD-10 elution and concentration by centrifugal filtration.

Supplementary Figures

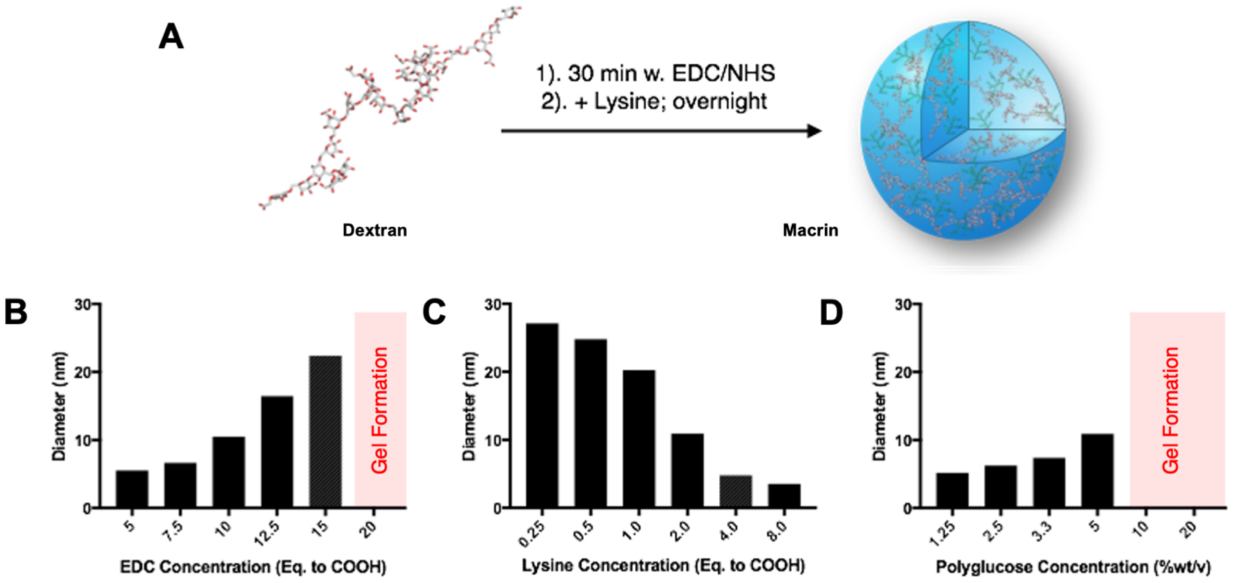

Figure S1. Control of macrin size through altered synthesis conditions. (A) Schematic of macrin nanoparticle synthesis. Control of macrin size through synthesis conditions, including **(B)** EDC feed ratio, **(C)** relative *L*-lysine crosslinker concentration, and **(D)** and the dextran concentration. General results are consistent with trends that may be expected, including an increase in particle size with increasing catalyst or substrate concentrations. Above a threshold value for each, formation of a solid and insoluble hydrogel was observed. Interestingly, *L*-lysine has a substantial effect on particle size; high concentrations likely result in the formation of lysine-grafted dextran and poly-*L*-lysine, whereas low concentrations may perpetuate the formation of larger but more less dense nanogel structures. The results demonstrate that macrin size may be easily tuned, such as by catalyst or crosslinker concentrations, to alter nanoparticle size, which is known to influence systemic half-life, macrophage uptake, tissue penetration, and ultimate biodistribution.

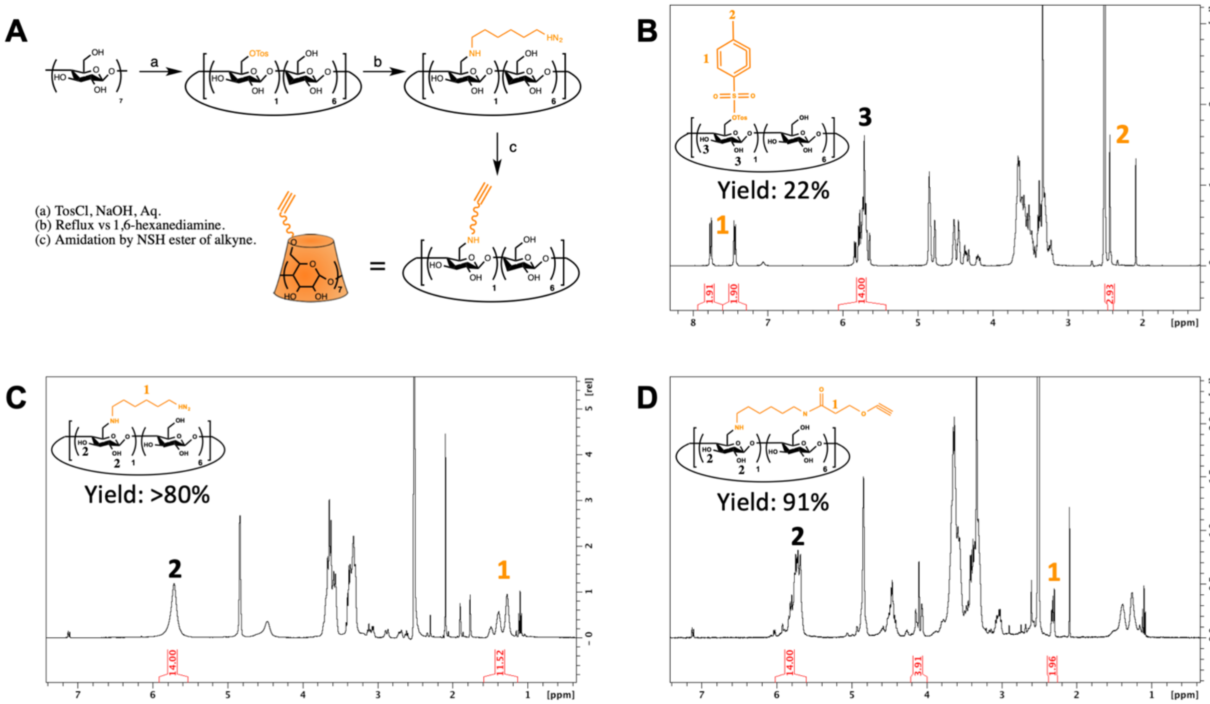

Figure S2. Alkyne modification of β-cyclodextrin. (A) Synthesis scheme for the preparation of alkyne-modified β-cyclodextrin. **(B-D)** ^{1}H-NMR spectra of intermediate compounds, including tosylated CD **(B)**, aminated CD **(C)** and the final propargyl CD **(D)**.

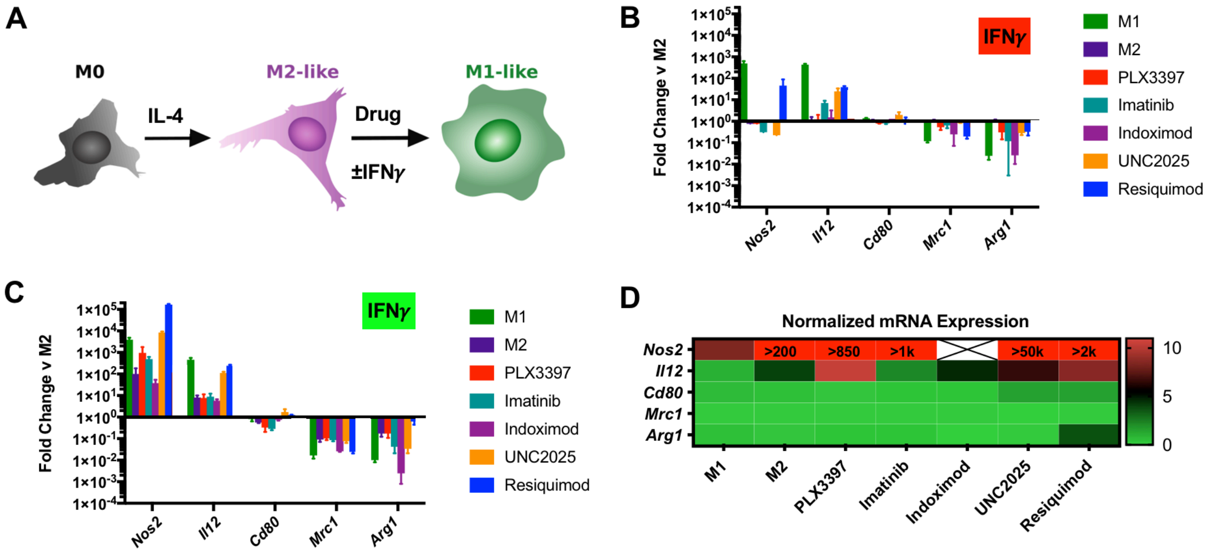

Figure S3. Transcriptional analysis of drug-treated macrophages. (A-C) The ability of drugs to induce M2-to-M1 re-education in the presence or absence of IFNγ was examined in IL-4 treated BMDMs. **(D)** The relative change in gene expression was heavily dependent on IFNγ, and only R848 consistently induced gene expression patterns indicative of an M1-like phenotype in its absence. Mean ± s.d., n=4 per condition.

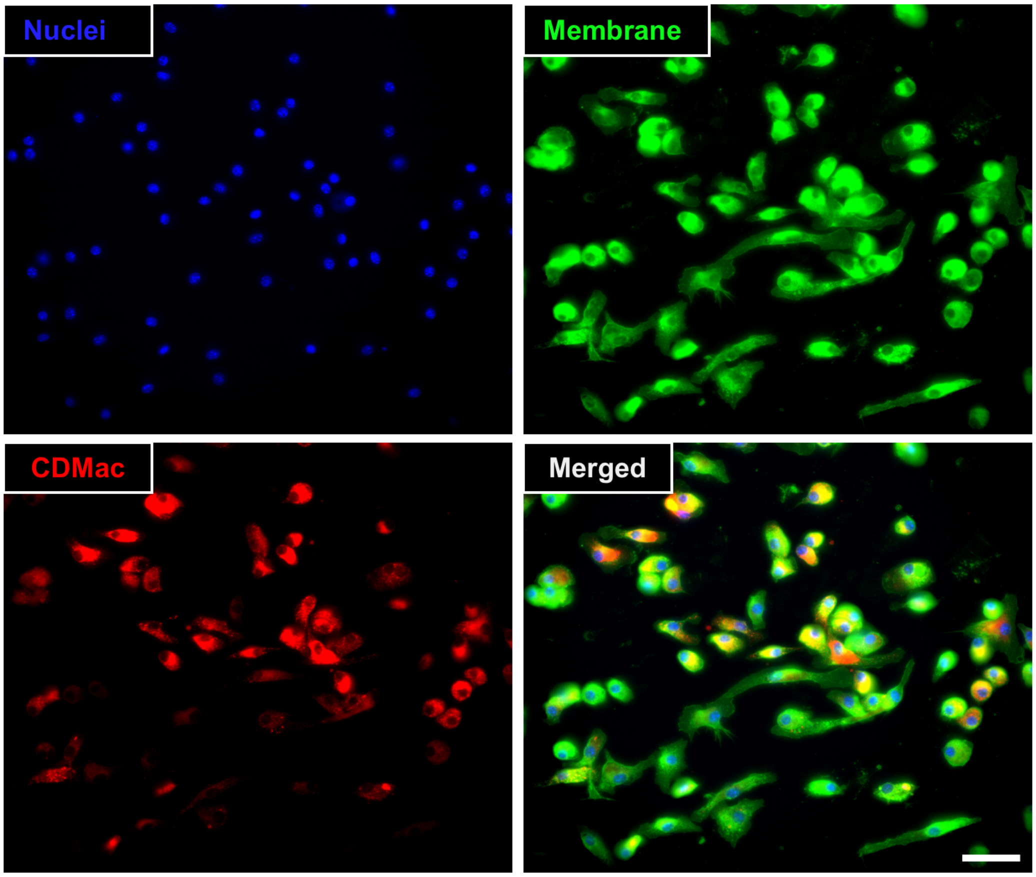

Figure S4. Expanded images of CDMac uptake. The uptake of CDMac-VT680 was examined by fluorescence microscopy in M2-like (IL-4 treated) BMDMs. Staining: DAPI (nuclei, blue); WGA-AF488 (cell membrane, green); CDMac-VT680 (nanoparticle, red). Scale bar: 50 µm.

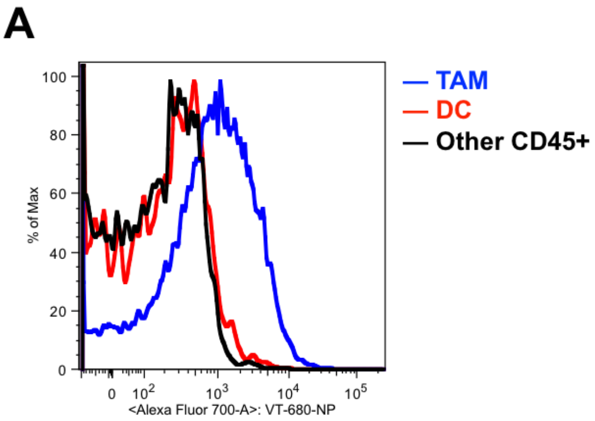

	Frequency (%CD45⁺)	MFI
TAM	69.9%	488
DC	5.44%	53
Other	19.5%	45.7

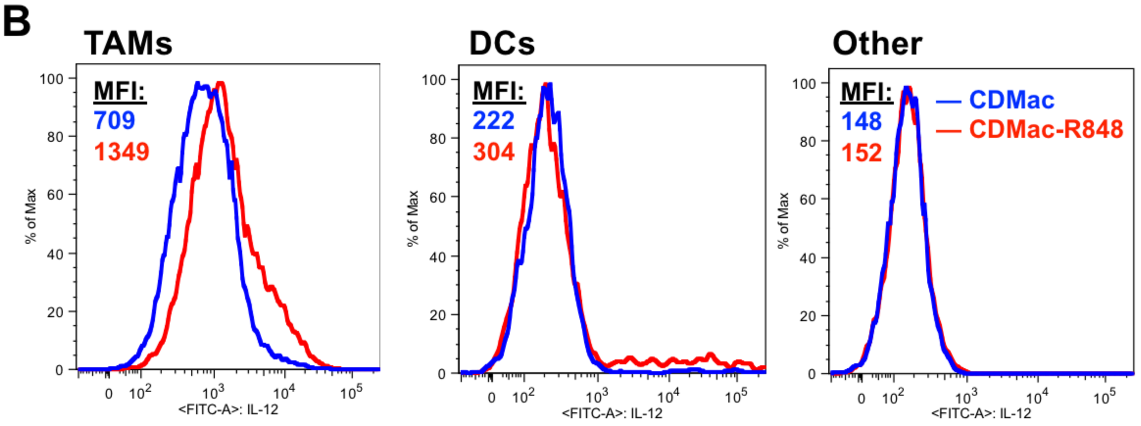

Figure S5. Flow cytometry of CDMac cellular biodistribution and IL-12 expression. MC38 tumors were harvested from p40-IRES-eYFP IL-12 reporter mice 48 h following tail vein injection of CDMac or CDMac-R848, each labeled by VT680. Flow cytometry was performed to identify macrophages ($CD45^{+}Ly6G^{-}F4/80^{+}$), dendritic cells ($CD45^{+}F4/80^{-}CD11c^{+}$), and other immune cells ($CD45^{+}F4/80^{-}$ $CD11c^{-}$). Fluorescence histograms demonstrated that (**A**) only TAMs (which make up nearly 70% of the local immune cell population) uptake CDMac, and (**B**) TAMs exhibit a near-twofold increase in production of IL-12-eYFP in response to CDMac-R848 (consistent with quantitative image analysis), while other immune cell populations contribute minimally to IL-12 production.

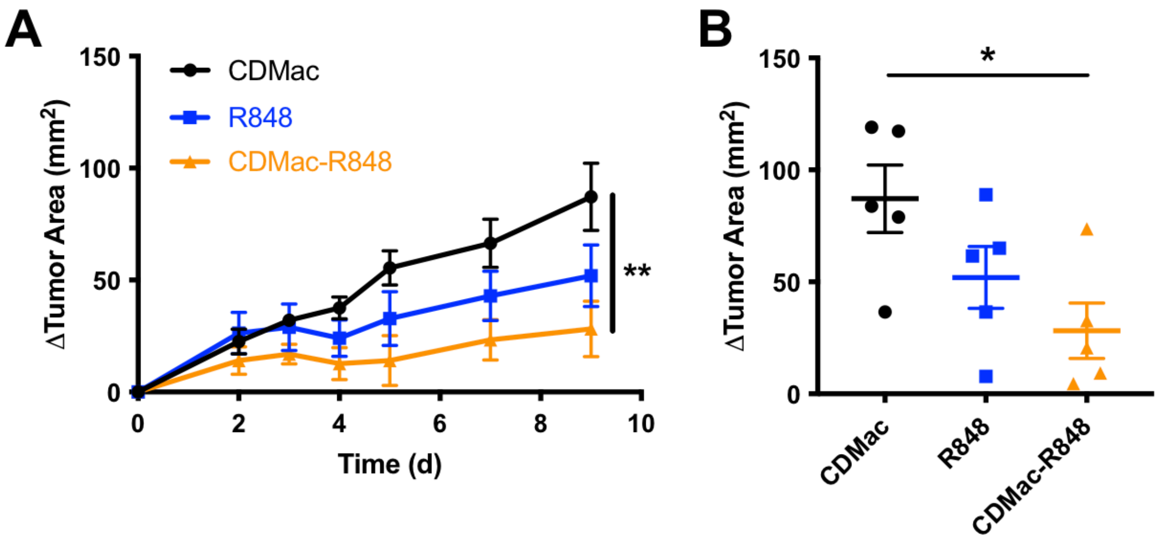

Figure S6. Mean tumor growth. (A) MC38 tumor growth curves. Mean ± s.e.m; n=5; **P<0.01 relative to CDMac controls; Friedman, Dunn's. **(B)** Change in tumor area at day 9, relative to animal baseline. Mean ± s.e.m; n=5; *P<0.05; Kruskal–Wallis, Dunn's.
